# Assessment of Knowledge about Traditional Medicine Reveals Overuse as a Potential Risk for Aggravating COVID-19 and Underlying Diseases in Geriatrics and Women’s Health in the Saudi Population

**DOI:** 10.3390/clinpract12030041

**Published:** 2022-05-23

**Authors:** Khalid Farhan Alshammari, Fadyah Mohammed Alradaddi, Kholah Fares Alshammari, Maha Qasem Almutairi, Nuseibah Saleh Almakhalfi, Raghad Abdullah Almeshari, Shamma Mutlaq Alaezaimee

**Affiliations:** 1 Department of Internal Medicine, College of Medicine, University of Ha’il, Hail 55476, Saudi Arabia; 2 College of Medicine, University of Ha’il, Hail 55476, Saudi Arabia; fadyah_1996@outlook.sa (F.M.A.); khawllahf20@gmail.com (K.F.A.); maha15@outlook.com (M.Q.A.); nuseibah_1997@outlook.sa (N.S.A.); rgd51283@gmail.com (R.A.A.); shama.alezaimee@gmail.com (S.M.A.)

**Keywords:** alternative medicine overuse, COVID-19 factors, Saudi medicinal and cultural practices

## Abstract

The devastating COVID-19 pandemic has created several gaps in the management of viral infections, leaving biocontainment and supportive measures as the only resorts for control. As such, there has been a dramatic increase in the use of dietary supplementations and herbal medicine for COVID-19. However, serious concerns regarding the efficacy, safety, and recommended doses of these medicines have been raised. In this study, we aimed to assess the population knowledge about alternative medicine administration for COVID-19 and the associated factors. Using a self-administered cross-sectional survey, we analyzed a total of 2042 valid responses. Most of the included participants were females (69.7%), with an overall mean age of 20.8 ± 11.8 years. Most respondents (62.8%) obtained their knowledge from social media while only 16.6% received knowledge from the health care workers. Half of the participants (50.6%) correctly identified all COVID-19 symptoms, where fever (18.5%) and loss of smell and taste (17.1%) were the most frequent answers. On the use of traditional medicines and supplements for COVID-19, 57.8% did not answer, 23.7% admitted regular use, and 18.5% used sometimes. Family members or friends suggested the use of traditional medicines and dietary supplements to 28.0% of the participants while only 14.7% were advised by a nutritionist, physician, pharmacist, nurse, or a health worker. Moreover, seniors and illiterate portions of society had lower knowledge scores and increased utilization of alternative medicine. Marital status, income, and previous COVID-19 were all significant predictors of the awareness and knowledge score. Thus, this study has identified overuse of unregulated medicinal products in the region, which potentially aggravates COVID-19 or other underlying risks of the disease, making clinical management challenging, particularly in geriatrics and women’s health. Regulation of medicinal products and establishment of educational campaigns about the disease have become imperative.

## 1. Introduction

The ongoing pandemic outbreak of severe acute respiratory syndrome (SARS-CoV2), the causative agent of COVID-19, has devastated global healthcare and economics. For nearly two consecutive years, COVID-19 has remained a clinical challenge, dramatically ravaging public health and wealth at an unprecedent speed. For instance, as of 21 October 2021, there have been 241,886,635 confirmed cases of COVID-19, including 4,919,755 deaths, reported to the World Health Organization (WHO), making it one of the most devastating, fastest, and deadliest coronavirus outbreaks in recent history (available at https://covid19.who.int/, accessed on 15 December 2021). Of all the vague scenarios the virus brought about, its complicated pathogenicity and epidemicity have created significant clinical challenges in the absence of a specific treatment. Control efforts were entirely focused on preventive and biocontainment measures [1,2]. Under these circumstances, in an unprecedented global effort, the birth of the most rapid, novel, and advanced state-of-the-art vaccine products in human history were recorded, including the Pfizer Biontech, Moderna, and AstraZeneca. This breakthrough has led to extensive vaccination campaigns ever witnessed. As of 20 October 2021, a total of 6,655,399,359 vaccine doses have been administered (available at https://covid19.who.int/, accessed on 15 December 2021). However, despite their high efficacy and safety, cases of potential side effects were reported. A prothrombotic syndrome in a small number of individuals after AstraZeneca vaccine administration was observed [3,4]. Unfortunately, these uncertainties have left the doors wide open for the uncontrolled consumption of alternative medicines encouraged by social media platforms and antivaccine campaigns.

Misconceptions about the efficacy of the medicinal plant extracts and other food supplement have suddenly loomed on the markets and over-the-counter shelves under circumstances of reduced compliance. Consequently, other interventional approaches against the infection were reported. Among these, herbal products have been claimed to be efficacious modalities that can enhance immunity and intervene against COVID-19 [5,6]. A previous report from the WHO indicated that the rate of utilization of herbal products is variable across different countries. While they are moderately consumed in some countries, they are always administered in others. The motive for this practice is based on many factors but primarily the cultural and habitual aspects of different societies [7].

Despite limited reports on the evidence for significant efficacy of these products against COVID-19, there has been increased utilization in different populations [5,6,8,9]. Besides, increased administration of raw products has the potential risk for adverse drug–drug interactions and significant pharmacodynamics and pharmacokinetics with other conventional drugs, reducing the efficacies of the latter medicines [10]. For instance, in Saudi Arabia, the utilization of high levels of natural raw products, such as honey, black cumin, dietary supplements, myrrh, ginseng, cinnamon, and ginger, across the different regions within the country occurred before the COVID 19 pandemic as shown by many studies [11,12,13]. This is probably attributable to the common attitude and satisfaction towards these products based on many aspects of local lifestyles and beliefs [12,14,15].

The high global consumption rate of dietary supplementations and herbal medicinal plants during the COVID-19 pandemic is worrisome [16,17,18,19]. This consumption is supported by reports on good outcomes from countries known historically to consume herbal extracts. Since these reports are intended for international audiences, an awareness against common use in different regions is imperative due to the differences in the population genetic structures of societies andlocal nutritional practices, and geographic differences in ecological factors. Thus, impressive correlations to well-being have been proven to lure certain layers of society into over use. For instance, studies that revealed an association between the utilization of herbal products in China with a significant decline in the rates of infections would be tempting to use (or misue) in different geographic regions globally [17]. In this context, a similar previous meta-analysis of randomized controlled trials reported that a combination of herbal medicine and conventional medications can significantly relieve the symptoms of COVID-19 [16]. However, a few similar studies conducted in Saudi Arabia on alternative medicinal use during the COVID-19 pandemic showed variable results [20,21,22]. For these reasons, it is important to increase the awareness and knowledge about the importance of the use of these products and the target society that it is likely to benefit. Thus, reports on the knowledge and awareness about herbal medicinal use during COVID-19 are limited in Saudi Arabia. In addition, the compatibility of medicinal plants is widely known to be dependent on the local genetic structures of residents. In other words, the diversity in the population genetic structures of different regions potentially plays a significant role in the compatibility of certain drugs. For these reasons, this study aimed to conduct and assess the knowledge and awareness of local alternative medicine administration against COVID-19 and its associated factors.

## 2. Materials and Methods

### 2.1. Study Design

The current study was conducted as a cross-sectional survey using a self-administered structured online questionnaire through an anonymous “SurveyMonkey” platform.

### 2.2. Study Population

All individuals who agreed to participate in the study who were aged ≥ 18 years and living in Saudi Arabia were eligible to participate. We imposed no restrictions on the gender, nationality, occupation, or socioeconomic level of the participants. Snowball sampling was used to select the study participants.

### 2.3. Data Collection

An online link to the web-based survey was developed using “SurveyMonkey” to obtain data regarding the use of alternative medicine in COVID-19, from January to April 2021. On the first screen of the questionnaire, a plain language information statement (PLIS) and consent form were enclosed. The contact details of the study investigators were included in the PLIS, who were able to respond to any relevant queries during data collection. Only the participants providing consent to participate in the study could move to the next section containing the screening questionnaire to confirm their age. If their age was consistent with the pre-defined range, the participants were moved to the next pages containing the self-administered survey [22].

### 2.4. Questionnaire Formulation and Validation

In the process of developing the questionnaire, an extensive review of the available literature was performed, followed by a discussion with experts. Following the development of the first version of the questionnaire, it was validated by a panel of experts regarding its face, content, criterion, and construct components. For further evaluation, a pilot study of 30 participants was performed, where different reliability measures were also tested, including test-retest reliability/repeatability (Pearson correlation), internal consistency (Cronbach’s alpha), and inter-rater reliability. The overall Cronbach’s Alpha was 0.89, which is higher than the minimum acceptable value (0.7).

### 2.5. Study Tool

The final format of the survey tool consisted of 20 questions, which were divided into 4 different sections: (1) personal data (7 questions: age, gender, region, education level, marital status, occupation, monthly income); (2) special habits (1 question: smoking); (3) medical history (2 questions: chronic diseases, COVID-19 history); and (4) knowledge and awareness of alternative medicine in COVID-19 prevention (10 questions). Participants’ answers for the last section were transferred into a score for easier interpretation and testing. For the sources of knowledge, only health care workers were given one point while other sources were considered unreliable. For symptom identification, one point was given for each symptom, with a total of eight points possible. For all other questions, support for using alternative medicine or supplements was scored −1, those who were against them were given 1 point, and neutral or hesitating options were scored zero. This was conducted to identify a direction for the score, where a higher score means higher knowledge and less support for the role of alternative medicines or supplements. Finally, the maximum score possible was 17 while −6 was the least possible.

### 2.6. Statistical Analysis

Data were analyzed using IBM SPSS for Windows version 26 statistical software(Chicago, IL, USA). Categorical data were reported as a frequency/percentage and continuous data as a mean/standard deviation. Continuous data were explored for normality by checking the distribution of data and using tests of normality (Kolmogorov–Smirnov and Shapiro–Wilk tests). The Chi2 test (or Fisher’s exact test, as appropriate) and independent t-test (or the Mann–Whitney U test as appropriate) were used for testing the difference based on the participants’ gender. Moreover, univariate linear regression was used to identify the possible predictors for the awareness and knowledge of alternative medicine.

## 3. Results

### 3.1. Sociodemographic Characteristics of the Participants

A total of 2042 valid responses were included in the analyses. Most of the respondents were females (69.7%), with an overall mean age of 20.8 ± 11.8 years. For regional distributions, the most respondents were from the central region (26.5%), followed by the western (25.1%), eastern (23.5%), and northern (18.6%) regions, respectively. Most of the participants had a university degree or higher (66.4%). In total, 28.4% had a high school diploma, 4% a middle school, and 1% had a higher school. Nearly half of the participants were married (49.9%) and had an income of less than 5000 SR per month while only 39.1% of them were employed. In total,49.9% were married with an income of less than 5000 but 39.1% were employe. Most of the participants were non-smokers (85.6%), did not suffer from any chronic conditions (86.3%), or had previously had COVID-19 (86.8%). There were statistically significant differences among males and females in terms of age (*p*-value < 0.001), residence (*p*-value < 0.001), educational level (*p*-value = 0.037), marital status (*p*-value = 0.012), employment status (*p*-value < 0.001), economic level (*p*-value < 0.001), and smoking habit (*p*-value < 0.001) (Table 1).

### 3.2. Awareness and Knowledge of Alternative Medicine

**Figure 1:**
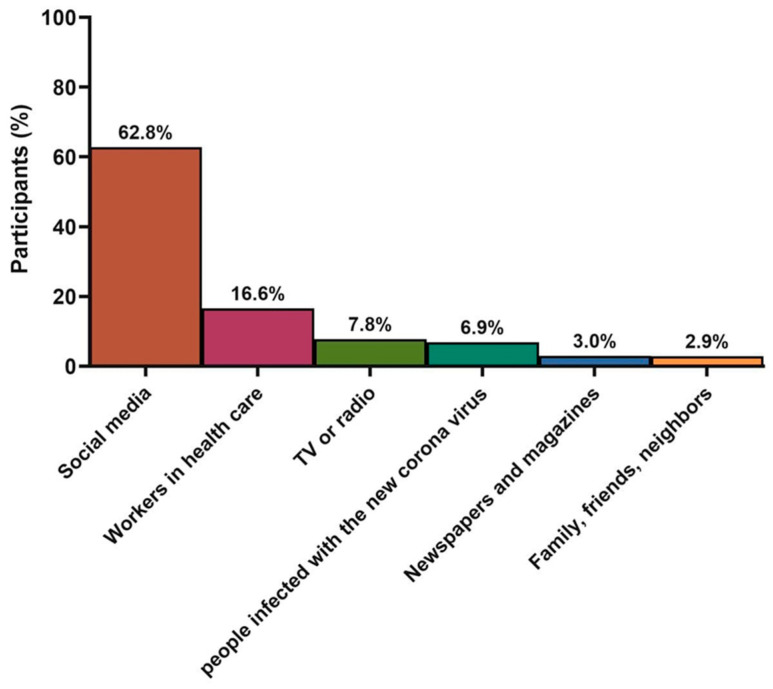
Sources of information about the novel coronavirus (COVID-19).

Most of the participants (62.8%) reported obtaining their information about COVID-19 from social media while only 16.6% were informed by health care workers. In addition, TV/radio (7.8%), previously infected people (6.9%), newspapers/magazines (3.0%), and family, friends, neighbors (2.9%) were all reported sources (Figure 1).

Half of the participants (50.6%) identified all symptoms of COVID-19 properly, with fever (18.5%) and loss of smell and taste (17.1%) being the most frequently reported; however, only 2% of the participants did not recognize any of the COVID-19 symptoms. About one-quarter (25.5%) of the participants did not think that traditional medicine and dietary supplements would prevent or reduce the odds of contracting COVID-19 while 51.5% of them thought it may, and 23.0% said it would. Most of the participants (70.5%) disagreed with the statement that traditional medicines and dietary supplements would protect against COVID-19 more than the social distancing. In terms of using traditional medicines and dietary supplements to prevent COVID-19, 57.8% of the participants did not use them, 23.7% regularly did, and 18.5% sometimes did. Family members and friends suggested the use of traditional medicines and dietary supplements to 28.0% of the participants while social media or other websites motivated 2.5%, and only 14.7% used those materials based on a piece of advice from a nutritionist, physician, pharmacist, nurse, or health worker (Table 2).

Dietary supplements were mostly used (40.9%); however, 31.4% reported using traditional medicines, 1.3% used yoga, and 1.7% used Chinese needles or cupping. Only 20.2% of the participants had consulted a doctor prior to using traditional medicines or dietary supplements. Regarding the efficacy, 30.3% thought that the materials used were effective to very effective in treating COVID-19. Nevertheless, an overwhelming majority of participants (87.0%) did not agree with the statement that using traditional medicines or dietary supplements can replace vising a doctor when COVID-19 is contracted. The percentage of those who did agree (13.0%) is still a considerable portion (Table 2).

The mean score of all participants was 4.8 ± 4.4 out of 17 possible points, which indicates a problem regarding the knowledge and support towards the use of traditional medicines or dietary supplements in COVID-19 prevention or treatment. Age was a significant predictor of the awareness and knowledge score (*p*-value < 0.001). Seniors were associated with less knowledge and were more likely to use traditional medicine. In contrast to university or higher education degree holders, the middle school (*p*-value < 0.001) and high school diploma holders (*p*-value = 0.033) had significantly lower knowledge scores and a high tendency to use traditional medicines or supplements (Table 3).

## 4. Discussion

The devastating COVID-19 pandemic has created significant gaps in clinical management strategies, opening several doors for alternative medicines. The consumption of herbal medicine and alternative health products has been widely reported globally, either to enhance health-related outcomes or relating to claims of relieving the symptoms and severity of disease conditions. These modalities are used in the absence of effective management strategies in cases of viral infections or other drug-resistant disorders where they have often proved efficacious. A compelling example of this is the current COVID-19 pandemic, for which no effective pharmacological modalities have been validated yet and vaccination does not rule out re-infection. However, at the time of submission of this manuscript, molnupiravir appeared in news headlines as the first oral antiviral COVID treatment (available at [https://www.nature.com/articles/d41586-021-02783-1], accessed on 22 October 2021 at 10:23 PM local time). The pandemic has caused serious health burdens, such as fear, anxiety, and panic, which has stimulated personal judgments on the use of alternative medicines. Food supplementations and herbal medicine are often suitable in such situations, provided they are used under medical supervision or advise, where favorable outcomes have been reported. The lack of suitable pharmacological modalities paralleled with the emergence of many drug-resistant pandemics has resulted in innovative approaches to the validation of herbal medicines. This was evident during the evolution of the SARS-1 and Middle East respiratory syndrome (MERS) pandemics. Widespread consumption of herbals in Saudi Arabia was reported by Alkhamaiseh et al. [23]. In the aforementioned study, 94% of adult Saudis used it for general therapeutic purposes and 54% as a first-line treatment in the case of diseases despite appearance of side effects in 46% of users. Furthermore, Saudis administer herbal medicine in certain circumstances, such as pregnancy [24] and diabetes [25], with previously estimated prevalence rates of 33% and 68%, respectively.

In the present study, we assessed the knowledge and attitudes regarding the use of herbal medicine during the COVID-19 pandemic. Our results showed that only around 23% of the study population thought that herbal medicines can reduce the chances of developing the COVID-19 infection. On the other hand, 70% believed that such treatment regimens are not better than social distancing in reducing the rates of COVID-19 infection. This is in agreement with a recent web-based study where public perceptions regarding social distancing during COVID-19 were high based on proper information from the right sources [26]. However, 23.7% of participants used herbal medicines during the COVID-19 pandemic for protection and prevention against the disease. This is consistent with a previous finding, where 22.1% of Saudis reported administration of herbal medicines [18]. On the other hand, AlNajrany et al. [19] reported that 64% of their population reported using herbal products during the COVID-19 pandemic for therapeutic and interventional purposes. Furthermore, Aldwihi et al. [27] indicated a significant increase in patients’ administration of food supplementations and herbal products post COVID-19 infection. The authors also reported that frequent administration of vitamin C, peppermint, and lemon or orange were significantly associated with a reduced odds of being hospitalized for severe COVID-19 infections. Many previous investigations have also reported on the use of known herbal medicine and food supplementation for therapeutic and preventive measures during pandemics [20,28,29,30].

Senior and non-college-educated participants were significantly less informed with lower knowledge scores, as indicated by their increased use of traditional medicine. We also found that marital status, income, and previous COVID-19 infection were all significant predictors of the knowledge and awareness scores among the target population. This is consistent with the finding of AlNajrany et al. [21], who found that elders and previous experience with the products was associated with significantly more frequent utilization of traditional medicine. Other investigations have reported increased knowledge and awareness of individuals with higher educational levels about preventive measures effective against COVID-19 [20,31,32]. Although issues on gender differences has been a controversial topic in different investigations [20,31], it was not significant in our study, which is probably due to the potential differences in the baseline demographics among the different studies and in the assessment parameters that were employed by each investigation. Moreover, previous studies have shown that much of their target population thought traditional medicine was safer and better in the management of many disorders than conventional medications [20,33,34]. Welz et al. [35] previously reported that family habits, cultural beliefs, feasibility of herbals, and their occasional good outcomes following administration reduced satisfaction with conventional drugs.

In recent decades, the steady increase in herbal use and the growing sense of belief over conventional medicine has raised questions, particularly in relation to frequent adverse reactions, incompatibilities, and challenges in monitoring safety [36]. There are many serious cases of herb–drug interactions where either the therapeutic efficacy of specific medications were lost or adverse events might have occurred [37]. These situations can affect the potential benefits of the drugs in reducing the severity of COVID-19 [16]. In this study, we showed that about 80% of the target population administer alternative medical products without any medical supervision. Thus, nationwide awareness and educational campaigns are recommended to increase the knowledge and awareness about the use and quantification of these products under medical supervision.

Our study is limited by the cross-sectional design and the online approach of collecting data and recruiting participants, which could be associated with selection bias. In addition, the sample is not representative of the whole population. There are also significant baseline differences between males and females, which is a potential confounder. Moreover, the estimated significant differences in the baseline characteristics among the recruited participants might also account for a potential impact on the reported outcomes. Therefore, future investigations are needed with better sampling.

## 5. Conclusions

In this study, we assessed the knowledge and awareness about the use of different types of traditional medicines in Saudi Arabia. This study revealed that the elderly, seniors, and uneducated participants were associated with lower knowledge scores and increased utilization of alternative medicine. On the other hand, marital status, income, and previous COVID-19 were all significant predictors of the awareness and knowledge score. In total, 62.8% of the participants relied on social media for knowledge compared to only 16.6% on knowledge from health care workers. On the use of traditional medicines and supplements for COVID-19, the majority (57.8%) did not answer, 23.7% admitted regular use, and 18.5% used sometimes. Moreover, family members played a significant (28.0%) role in persuading participants to use such products while only 14.7% were advised by a nutritionist, physician, pharmacist, nurse, or health worker. Thus, educational campaigns should target portions of the population to provide proper knowledge on the administration and benefits of alternative medicines in addition to an awareness of the specific benefits of conventional drugs and their intended use over herbs.

## Figures and Tables

**Table 1:** Sociodemographic characteristics of the participants.

Variables		Gender						*p*-Value
		Female		Male		Total		
		Count	%	Count	%	Count	%	
Age (years); mean ± SD		19.7 ± 11.1		23.3 ± 128		20.8 ± 11.8		<0.001 *
Residence (region)	Central	362	25.4	179	28.9	541	26.5	<0.001 *
	Eastern	310	21.8	169	27.3	479	23.5	
	Northern	243	17.1	137	22.1	380	18.6	
	Southern	100	7.0	30	4.8	130	6.4	
	Western	408	28.7	104	16.8	512	25.1	
Educational level	Primary School	18	1.3	3	0.5	21	1.0	0.037 *
	Middle school	66	4.6	15	2.4	81	4.0	
	High school diploma	390	27.4	190	30.7	580	28.4	
	University degree or higher	946	66.5	410	66.2	1356	66.4	
	None	3	0.2	1	0.2	4	0.2	
Marital status	Divorced	46	3.2	14	2.3	60	2.9	0.012 *
	Married	688	48.3	331	53.5	1019	49.9	
	Single	669	47.0	273	44.1	942	46.1	
	Widow	20	1.4	1	0.2	21	1.0	
Employment status	Employed	419	29.4	380	61.4	799	39.1	<0.001 *
	Unemployed	1004	70.6	239	38.6	1243	60.9	
Monthly income	5000–10,000 SR	308	21.6	127	20.5	435	21.3	<0.001 *
	Less than 5000 SR	868	61.0	220	35.5	1088	53.3	
	More than 10,000 SR	247	17.4	272	43.9	519	25.4	
Do you smoke?	No	1360	95.6	388	62.7	1748	85.6	<0.001 *
	Yes	63	4.4	231	37.3	294	14.4	
Do you suffer from chronic diseases?	No	1241	87.2	521	84.2	1762	86.3	0.066
	Yes	182	12.8	98	15.8	280	13.7	
Have you been infected with COVID-19?	No	1234	86.7	539	87.1	1773	86.8	0.826
	Yes	189	13.3	80	12.9	269	13.2	

* *p*-value < 0.05 is significant.

**Table 2:** Awareness and knowledge of alternative medicine.

Variables		Count	%
What are the symptoms of the novel Corona virus?	Cough	40	2.0
	Diarrhea	82	4.0
	Fever	378	18.5
	Headache	37	1.8
	Lethargy	14	0.7
	Loss of smell and taste	350	17.1
	Shortness of breath	61	3.0
	Vomiting	9	0.4
	All of them	1034	50.6
	None of them	37	1.8
Do you think that the use of traditional medicine and Vitamins prevents or reduce the odds of the novel Corona virus infection?	Maybe	1052	51.5
	No	520	25.5
	Yes	470	23.0
Do you think that traditional medicines and dietary supplements protect against corona virus more than social distancing?	Maybe	449	22.0
	No	1440	70.5
	Yes	153	7.5
Do you use any type of alternative medicine and dietary supplements to prevent infection with the new corona virus?	No	1180	57.8
	Sometimes	378	18.5
	Yes	484	23.7
If yes, who suggested you take a dietary supplement or a traditional medicine?	Family member or Friends	571	28.0
	Nutritionist/physician/pharmacist/nurse/health worker	301	14.7
	Social media and other websites	479	23.5
	Did not use	691	33.8
If yes, which type of supplements do you use?	Traditional medicines	641	31.4
	Dietary supplements	836	40.9
	Chinese needles and cupping	34	1.7
	Yoga	87	4.3
Have you consulted a doctor before using traditional medicines or nutritional supplements?	No	1630	79.8
	Yes	412	20.2
Do you think that folk medicines or nutritional supplements can replace visiting a doctor in case of infection with the novel Corona virus?	No	1777	87.0
	Yes	265	13.0
How effective are traditional medicines and nutritional supplements in treating the novel Corona virus?	Effective	505	24.7
	Ineffective	427	20.9
	Neutral	996	48.8
	Very effective	114	5.6

**Table 3:** Univariate linear regression of different predictors of the awareness and knowledge score.

Predictor	Estimate	SE	t	*p*-Value	Standardized Estimate	95% Confidence Interval	
						Lower	Upper
Age (years)	−0.07	0.01	−8.64	<0.001 *	−0.19	−0.23	−0.15
Gender							
Female	*Reference*						
Male	0.33	0.21	1.52	0.128	0.07	−0.02	0.17
Residence							
Central	*Reference*						
Eastern	−0.32	0.28	−1.155	0.248	−0.07	−0.20	0.05
Northern	0.03	0.30	0.112	0.911	0.01	−0.12	0.14
Southern	0.44	0.43	1.027	0.305	0.10	−0.09	0.29
Western	−0.56	0.27	−2.06	0.039	−0.13	−0.25	−0.01
Educational level							
University degree or higher	*Reference*						
High school diploma	−0.47	0.22	−2.13	0.033 *	−0.11	−0.20	−0.01
Middle school	−1.76	0.51	−3.48	<0.001 *	−0.40	−0.62	−0.17
Primary school	−1.44	0.97	−1.48	0.14	−0.32	−0.75	0.11
None	−2.81	2.22	−1.27	0.205	−0.63	−1.61	0.35
Marital status							
Single	*Reference*						
Divorced	−1.17	0.58	−2.01	0.045 *	−0.26	−0.52	−0.01
Married	−1.52	0.2	−7.7	<0.001 *	−0.34	−0.43	−0.26
Widow	−2.42	0.97	−2.5	0.012 *	−0.54	−0.97	−0.12
Employment							
Employed	*Reference*						
Unemployed	0.12	0.2	0.61	0.542	0.03	−0.06	0.12
Income							
Less than 5000 SR	*Reference*						
5000–10,000 SR	−0.86	0.25	−3.42	<0.001 *	−0.19	−0.3	−0.08
More than 10,000 SR	−0.33	0.24	−1.4	0.161	−0.07	−0.18	0.03
Smoking	−0.13	0.28	−0.47	0.636	−0.03	−0.15	0.09
Chronic diseases	−0.16	0.29	−0.57	0.566	−0.04	−0.16	0.09
Previous COVID−19	−0.94	0.29	−3.23	0.001 *	−0.21	−0.34	−0.08

SE standard error; * Statistically significant.

## Data Availability

All data related to this article is embedded within the manuscript. For the survey used, data can be shared by direct contact of the corresponding author.
